# Professor T. R. Beck

**Published:** 1854-07

**Authors:** 


### PROFESSOR T. R. BECK.

This eminent writer and physician has relinquished the direction of the *Journal of Insanity*, from the pressure of increasing years, and of other incumbent duties. His friends will regret to learn that he begins to feel the weight of age. He has the best wishes of all his countrymen, that the vigor of his faculties may long remain to him for the prosecution of those labors by which he has added so much to the reputation of his country.

